# Letter in response to ‘Research papers by radiographers and radiation therapists: An Australian perspective’

**DOI:** 10.1002/jmrs.35

**Published:** 2014-01-06

**Authors:** Beverly A Snaith

**Affiliations:** Radiology Department, Mid Yorkshire Hospitals NHS Trust, Pinderfields HospitalAberford Road, Wakefield, WF1 4DG, United Kingdom

Re: Snaith B. An evaluation of author productivity in international radiography journals 2004–2011. J Med Radiat Sci 2013; 60(3): 93–9

I appreciate the opportunity to respond to the comments of Dr Schneider and agree that this article represents only a limited representation of radiography authorship. However, in a research active profession the debate is important and she acknowledges radiography-specific journals are not seen as overly attractive to authors. As an author who publishes in a wide range of journals I understand the problem personally. The difficulty remains at tracking radiographer authors through the wider literature and therefore discipline-specific journals remain our core evidence of research activity as a profession.

I expect *JMRS*, building on its predecessor journals, hopes to attract high-quality articles, and an understanding of the current picture is important in planning for both the journal and professional bodies. The requirement for many researchers to publish in impact factored journals remains an issue for all included within this study and is underpinned with a need to demonstrate a unique evidence base. This is where the profession-specific journals should have an opportunity, but if submissions, particularly at an international level, and reject rates are low, Medline indexing (and a subsequent impact factor) will remain an aspiration. The comment regarding Scopus indexing is an error and should be Medline index, as Dr Schneider recognised that none of the journals is listed.

This article was aimed at providing an understanding of the current evidence of radiography authorship, and as such it is hoped that the debate will in future continue to be ‘where to submit’ rather than ‘whether to write’.

